# Exploring the Novel Pancreatic Lipase-Inhibitory Peptides in *Chlorella pyrenoidosa*: Preparation, Purification, Identification, and Molecular Docking

**DOI:** 10.3390/foods14183277

**Published:** 2025-09-22

**Authors:** Dengmi Wang, Luan Lin, Peng Liang, Wenjun Liu, Wenrui Ma, Ying Wang, Jicheng Chen

**Affiliations:** 1 Fujian Province Key Laboratory for the Development of Bioactive Material from Marine Algae, College of Oceanology and Food Science, Quanzhou Normal University, Donghai Street 398, Quanzhou 362000, China; 52309010051@fafu.edu.cn (D.W.); liangpeng137@sina.com (P.L.); lwj08162025@163.com (W.L.); 18993418056@163.com (W.M.); 19959708276@163.com (Y.W.); 2 College of Food Science, Fujian Agriculture and Forestry University, Shangxiadian Road 15, Fuzhou 350001, China; newtaicjc@163.com; 3 Key Laboratory of Inshore Resources Biotechnology, Fujian Province University, Quanzhou 362000, China

**Keywords:** *Chlorella pyrenoidosa*, pancreatic lipase-inhibitory peptides, purification, identification, molecular docking

## Abstract

Obesity is a worldwide problem, and lowering pancreatic lipase (PL) activity is an effective strategy to counteract it. In this study, pancreatic lipase-inhibitory (PL-I) peptides were isolated and purified from *Chlorella pyrenoidosa* protein hydrolysates (CPPHs) using ultrafiltration and Sephadex gel chromatography (Sephadex G-25). A total of 858 peptides were identified by liquid chromatography–tandem mass spectrometry (LC-MS/MS). Four novel PL-I peptides (FLSQPF, VWTPI, IVGPF, and IPYPL) were virtually screened using molecular docking and subsequently synthesized, with VWTPI exhibiting the highest PL inhibition. Moreover, the inhibition of the enzyme by VWTPI was a mixture of competitive and non-competitive inhibition, with an inhibition constant (*Ki*) of 7.27 mg/mL. Molecular docking showed that VWTPI interacts with the PL active center by hydrogen bonding, hydrophobic contacts, van der Waals forces, and π-π stacking. This study suggests that peptides from *Chlorella pyrenoidosa* could be used as lipid-lowering agents to prevent and cure obesity.

## 1. Introduction

Obesity has become a global problem and is often considered a risk factor for multiple diseases, such as diabetes and cardiovascular disease [1]. According to statistical data, the age-adjusted prevalence of adult obesity in China reached 42% from 2017 to 2018. It is projected that by 2030, 58% of the global adult population will be overweight or obese [2]. These alarming figures have raised substantial concerns and prompted increased attention toward the prevention and management of overweight and obesity in recent years. A major contributing factor to obesity is the adoption of unhealthy dietary habits and lifestyles, particularly the long-term consumption of high-fat diets [3]. Pancreatic lipase (PL) is a key enzyme responsible for the digestion and absorption of dietary fats [4,5]. Therefore, targeting pancreatic lipase to decrease fat digestion and absorption represents a promising strategy to combat obesity [6]. Furthermore, some studies have demonstrated a link between obesity and oxidative stress; antioxidant compounds have been shown to ameliorate lipid metabolism disorders, attenuate oxidative damage, and improve metabolic profiles in high-fat diet-induced obese rats, thereby reducing hepatic fat accumulation [7,8]. Currently, the commonly prescribed weight-loss and lipid-lowering drugs include orlistat, liraglutide, and statins, which are associated with certain side effects, including gastrointestinal adverse reactions, oily feces, fecal urgency, and oily spotting [9]. As a result, their widespread application remains limited. In contrast, bioactive peptides exhibiting pancreatic lipase-inhibitory activity have attracted interest as novel inhibitors due to their natural origin, high efficacy, and favorable safety profile, positioning them as promising therapeutic candidates.

Bioactive peptides (BAPs) are protein fragments, typically consisting of 2–20 amino acid residues, which remain inactive within their parent protein sequences and require liberation via enzymatic hydrolysis, fermentation, or chemical synthesis to exert diverse physiological functions [10,11]. Their mechanisms of action are closely linked to a high hydrophobic amino acid content. Although their structure–activity relationships have not been fully elucidated, BAPs possess notable advantages, including high bioactivity, low toxicity, low allergenic potential, and ease of metabolism, highlighting their significant potential in the prevention and management of chronic diseases [12]. In particular, food-derived BAPs have garnered increasing research interest in the field of lipid-lowering owing to their favorable safety and efficacy profiles. For example, the peptide FYLGYCDY, derived from rice bran fat extract, has been shown to effectively inhibit pancreatic lipase (PL) (IC50 = 0.47 ± 0.02 μM), suggesting its potential application as a weight-loss drug [13]. Similarly, the oat bran-derived peptide SPFWNINAH PL-I’s activity indicates its promise as an anti-obesity drug [14]. The peptide LLVVYPWTQR, isolated from *Chlorella*, has demonstrated efficacy in inhibiting pancreatic lipase and reducing intracellular triglyceride accumulation [15].

*Chlorella*, a form of single-cell green algae, contains over 50% protein and has become an important source of high-yield proteins [16]. Several species are commonly studied, including *Chlorella vulgaris*, *Chlorella ellipsoidea*, and *Chlorella pyrenoidosa*. Among these, *Chlorella pyrenoidosa* has attracted particular research interest due to its high nutritional value and was approved as a novel food resource in China in 2012 [17]. Peptides derived from *Chlorella* have been reported to exhibit various bioactive properties, such as antioxidant, anti-inflammatory [18], and antitumor activities [19]. However, to date, there is limited research specifically on lipid-lowering peptides derived from *Chlorella*. Therefore, this study used *Chlorella pyrenoidosa* protein as a substrate to prepare bioactive peptides through enzymatic hydrolysis. This approach aims to maximize the diversity of peptide bioactivities and discover novel peptide products with potential lipid-lowering functions.

Many recent studies have focused on the development of anti-obesity drugs using natural ingredients. However, few studies have reported the lipid-lowering effects of *Chlorella pyrenoidosa*. In this study, we aimed to isolate and purify PL-I peptides from the *Chlorella pyrenoidosa* protein hydrolysates. The peptide sequence was identified using LC-MS/MS, and its inhibitory effect on pancreatic lipase activity was evaluated. The results of this work can provide essential theoretical support for the development and application of natural PL-I peptides derived from *Chlorella*, and contribute to innovation and development of the *Chlorella* industry.

## 2. Materials and Methods

### 2.1. Materials and Chemicals

*Chlorella pyrenoidosa* in the form of spray-dried powder was purchased from Fuqing New Daze Spirulina Co. (Fuzhou, China). Alcalase (270,000 U/g, stored at 4 °C) was obtained from Novozymes Biotech Co., Ltd. (Tianjin, China). Pancreatic lipase (derived from porcine pancreas, 30,000 U/mg, purity ≥95%, and stored at −20 °C) was purchased from Yuanye Biotech (Shanghai, China). DPPH, 2,2-diazobis(3-ethyl-benzothiazole-6-sulfonic acid) diammonium salt (ABTS), and orlistat were purchased from McLean Biochemicals Ltd. (Shanghai, China) and stored at 4 °C, protected from light. All other chemicals and reagents used were of analytical grade and supplied by Xilong Science Co., Ltd. (Guangzhou, China).

### 2.2. Preparation of Chlorella Pyrenoidosa Protein Hydrolysates (CPPHs)

*Chlorella* protein powder (a defatted protein concentrate) was hydrolyzed using Alcalase at an enzyme-to-substrate ratio of 6000 U/g. The hydrolysis was performed in deionized water at a solid-to-liquid ratio of 15:1 (*w*/*v*, mg/mL), under the following conditions: pH 7.5, temperature 60 °C, and duration 5 h. The reaction was terminated by heating the mixture at 100 °C for 10 min. Subsequently, the hydrolysate was centrifuged at 8000 rpm for 15 min. The supernatant was collected, frozen at −80 °C, and then lyophilized using a freeze-dryer (Labconco, Kansas, MO, USA) for 48 h at a condenser temperature of −50 °C and a pressure of 0.1 mbar. The freeze-dried powder was stored at −20 °C for further analysis.

### 2.3. Ultrafiltration

Three fractions of varying molecular weights (>10 kDa, 10–5 kDa, and <5 kDa) were isolated by fractionating the CPPH using a laboratory-scale ultrafiltration system equipped with polyethersulfone (PES) membranes (model Pellicon^®^ 2, Sartorius, Göttingen, Germany) with molecular weight cut-offs of 10 kDa and 5 kDa. Before ultrafiltration, the hydrolysate was filtered through a 0.45 μm microfiltration membrane to remove suspended particles. The process was carried out at a constant temperature of 25 °C under an operating pressure of 0.3 MPa, with each fractionation step lasting approximately 90 min. Each resulting fraction was collected and freeze-dried for subsequent use.

### 2.4. Sephadex G-25 Gel Chromatography

The gel chromatography injection valve was cleaned with deionized water before sample injection and separation. The samples were analyzed following the protocol described by Shao et al. [20]. A manually operated glass column (1.6 × 70 cm) packed with Sephadex G-25 was used for separation. The sample was loaded at a concentration of 20 mg/mL with an injection volume of 2 mL. Elution was performed using ultrapure water at a flow rate of 1 mL/min, and the detection wavelength was set to 220 nm. The eluates at the same peak were collected, concentrated by a rotary evaporator, and freeze-dried.

### 2.5. PL Inhibition Assay

To evaluate the inhibitory activity of the peptide fractions against PL, we conducted experiments according to Zhang et al. [21]. Briefly, a mixture containing 120 μL of PL (10 mg/mL), 40 μL of peptide sample, and 80 μL of 50 mmol/L Tris-HCl buffer (pH 8.0) was incubated at 37 °C for 10 min. Then, 160 μL of 0.8 mmol/L p-nitrophenyl palmitate (pNPP) dissolved in dimethyl sulfoxide was added to initiate the reaction. After incubation at 37 °C for 20 min, the reaction was terminated by heating in a water bath at 100 °C for 5 min, followed by centrifugation to remove precipitated material. The absorbance of 200 μL of the supernatant was measured at 405 nm using a microplate reader (Infinite^®^ 200 PRO, Tecan, Männedorf, Switzerland). For the control group, the peptide sample was replaced with an equal volume of buffer; in the blank group, the PL solution was replaced with buffer. Finally, the activity of PL was calculated using Equation (1).

(1) $$PL\;activity\left(\%\right)=[1-\left(B-b\right)/(A-a)]\times 100\%$$

where A denotes the absorbance of the control group, B the absorbance of the sample group, a the absorbance of the control blank group, and b the absorbance of the sample blank group.

### 2.6. Evaluation of Antioxidant Activity

The samples of each fraction obtained after isolation and purification were dissolved in deionized water and configured into a peptide concentrate at a concentration of 16 mg/mL. The antioxidant activities, including DPPH radical scavenging activity [22], ·OH radical scavenging activity [23], and ABTS radical scavenging activity [24], were evaluated according to the established methods. A concentration series of each fraction was prepared to generate dose–response curves. The half-maximal inhibitory concentration (IC_50_) values were determined by linear regression analysis of the dose–response data. Vitamin C (VC) was used as a positive control in all three antioxidant assays. Based on the IC_50_ values, the fractions demonstrating the strongest antioxidant activity were selected for subsequent analysis.

### 2.7. Peptide Sequencing and Analysis

Peptide separation and analysis were conducted by Weinafei Biotechnology Co., Ltd. (Shenzhen, China) using an EASY-nLC 1200 system (Thermo Fisher Scientific, Waltham, MA, USA) coupled online with a Q Exactive mass spectrometer (Thermo Fisher Scientific, USA) equipped with a NanoFlex ion source. Trapping and desalting were performed with 20 µL of 100% solvent A (0.1% formic acid in water). Separation was achieved using a linear gradient from 5% to 38% solvent B (80% acetonitrile with 0.1% formic acid) over 30 min on an analytical column (Acclaim PepMap RSLC, 75 µm × 25 cm, C18, 2 µm, 100 Å, Thermo Fisher Scientific, Waltham, MA, USA). The spray voltage was set to 1.9 kV, the capillary temperature was maintained at 275 °C, and the mass spectrometry scan range was *m*/*z* 100–1500. Raw data obtained from the mass spectrometer were analyzed using PEAKS Studio 8.5 software (Bioinformatics Solutions Inc., Waterloo, ON, Canada). Database searching was performed against *Chlorella pyrenoidosa* from the UniProt database (https://www.uniprot.org/).

### 2.8. Molecular Docking

In this study, a modified version of Trott and Olson’s technique was applied [24]. The three-dimensional crystal structure of human pancreatic lipase (PL, PDB ID: 1LPB) was obtained from the RCSB Protein Data Bank (https://www.rcsb.org/). Protein pretreatment was conducted using AutoDockTools 1.5.7, which involved the removal of water molecules and natural ligands, followed by the addition of polar hydrogen atoms. Peptide structural models were constructed using ChemDraw 16.0.0. Molecular docking simulations between PL and VWTPI were performed using AutoDock Vina, considering ligand flexibility. The conformation with the lowest binding energy was chosen as the ideal binding mode for interaction analysis. The resulting interactions were visualized with PyMOL 3.0.0.

### 2.9. Solid-Phase Synthesis of Peptides

Peptides extracted from *Chlorella pyrenoidosa* are produced with the Fmoc solid-phase synthesis method [25] by Shangon Biotech Co., Ltd. (Shanghai, China). The purity and identity of each synthetic peptide were verified using high-performance liquid chromatography (HPLC) and electrospray ionization quadrupole mass spectrometry (ESI-QMS). HPLC analysis was performed on an Agilent 1260 system equipped with a C18 column (4.6 × 250 mm, 5 µm). Gradient elution was applied with mobile phase A (0.1% trifluoroacetic acid in water) and mobile phase B (0.1% trifluoroacetic acid in acetonitrile), ranging from 5% to 60% B over 30 min at a flow rate of 1.0 mL/min, with detection at 214 nm. Mass spectrometry was carried out in positive ion mode with a scan range of *m*/*z* 100–1500. All peptides were confirmed to have a purity of ≥95%. The inhibitory effect of these synthetic peptides on PL activity and their mechanism of inhibition were further investigated.

### 2.10. Kinetic Study of PL Inhibition

To investigate the inhibitory mechanism of the peptide on the enzyme, we evaluated its effect on enzymatic activity under different substrate concentrations (1, 2, 4, and 8 mmol/L) and peptide concentrations (0, 1, and 2 mg/mL). The initial reaction rates were measured and plotted against the enzyme concentration to preliminarily determine the inhibition type: a straight line passing through the origin with a reduced slope in the presence of the inhibitor suggested reversible inhibition. Further analysis was performed using Lineweaver–Burk double-reciprocal plots, where the inverse of the reaction rate (1/V) was plotted against the inverse of the substrate concentration (1/[S]). The mode of inhibition was identified by comparing changes in Km and Vmax across different inhibitor concentrations: unchanged Km and decreased Vmax indicated non-competitive inhibition; increased Km and unchanged Vmax suggested competitive inhibition; while an increase in Km accompanied by a decrease in Vmax indicated mixed inhibition [26]. Finally, the inhibition constant Ki was determined by constructing a secondary plot of the slopes from the double-reciprocal curves against the inhibitor concentration [26].

### 2.11. Statistical Analysis

All experiments were independently replicated three times (*n* = 3). Statistical analyses and data visualizations were carried out using GraphPad Prism 9.0 (GraphPad Software, Boston, MA, USA), Microsoft Excel 2021 (Microsoft Corporation, Washington, DC, USA), and OriginPro 2024 (OriginLab Corporation, Northampton, MA, USA). Significant differences in the graphs are marked with asterisks: * *p* < 0.05, ** *p* < 0.01 (two-tailed *t*-test or one-way ANOVA).

## 3. Results and Discussion

### 3.1. PL-I Activity of Ultrafiltration Fractions

**Figure 1:**
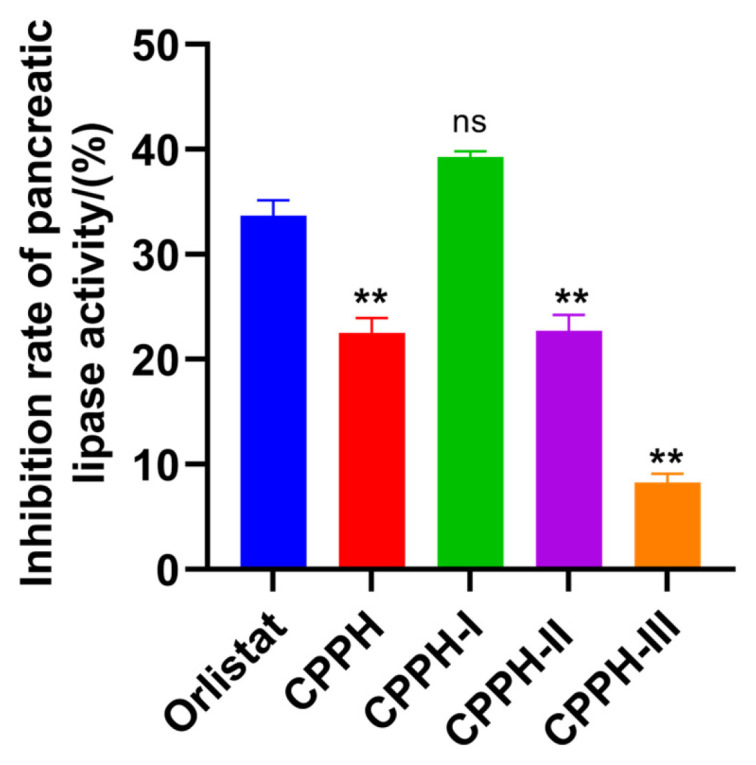
PL inhibition by ultrafiltration fraction. ** indicates *p* < 0.01; ns indicates no significant difference.

Given the significance of the molecular weight of protein hydrolysates in the synthesis of bioactive peptides, ultrafiltration is employed to separate the hydrolysate into several fractions with different molecular weights. It is important to note that ultrafiltration separation is not absolute; peptides near the molecular weight cut-offs may distribute across adjacent fractions, which is an inherent limitation of this technique that could influence the interpretation of bioactivity trends. Despite this limitation, ultrafiltration was employed as a standard initial fractionation step due to its scalability, operational simplicity, and effectiveness in providing a gross separation based on molecular size [27,28]. In this study, CPPH was selected for further fractionation and ultrafiltration separation into CPPH-I (<5 kDa), CPPH-II (5–10 kDa), and CPPH-III (>10 kDa). As shown in Figure 1, the inhibitory effects of CPPH and its fractions on PL activity were observed at a mass concentration of 8 mg/mL; the PL-I activity was clearly dependent on the molecular weight. CPPH-I exhibited the strongest PL-I activity (39.89 ± 0.54%), whereas CPPH-III had the weakest inhibitory effect on PL (8.26 ± 0.85%). There was no significant difference between CPPH-I and orlistat (at 8 µg/mL) (*p* > 0.05). In general, peptides with lower molecular weights have higher inhibitory activity [28]. Consistent with Zhang et al. [15], peptides with molecular weights <5 kDa exhibited significantly stronger PL-I activity than their higher-molecular-weight counterparts. Similarly, Ketprayoon et al. [10] isolated five molecular weight fractions via ultrafiltration from Alcalase-catalyzed hydrolysates of de-oiled rice bran and identified the <0.65 kDa fraction as the most potent lipase inhibitor. Smaller-molecular-weight peptides exhibit higher PL-I activity, which may be attributed to their greater bioavailability, easier interaction with the active site of pancreatic lipase, and increased hydrophobic amino acid content, which facilitates binding to the hydrophobic surface of the enzyme. Given its prominent inhibitory activity, CPPH-I was selected for further isolation and purification, providing a foundation for subsequent in-depth research and potential applications.

### 3.2. Antioxidant Activity Evaluation of Ultrafiltration Fractions

**Figure 2:**
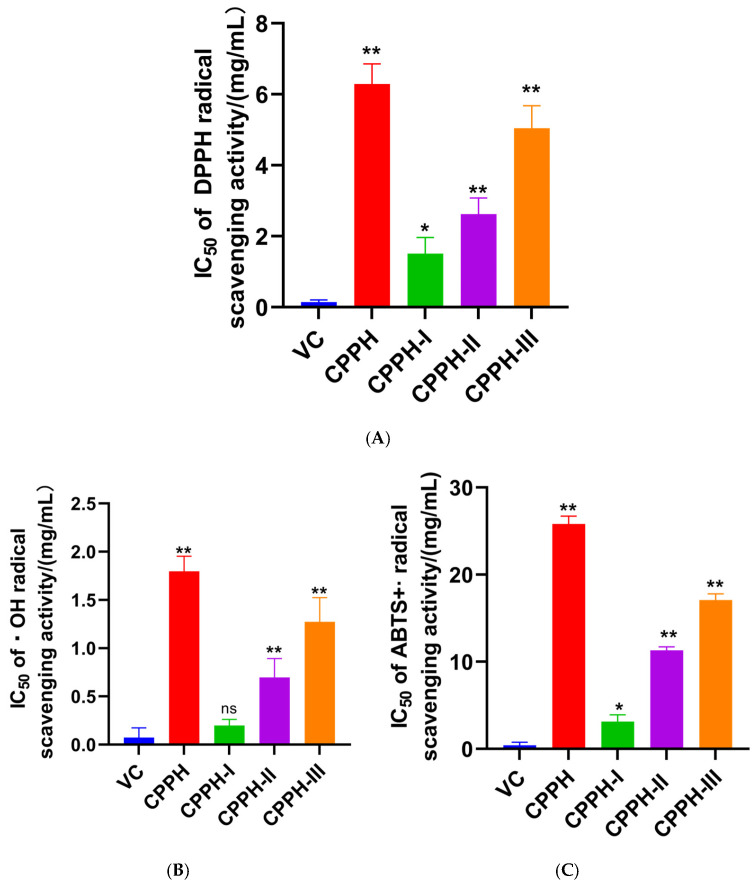
Antioxidant activity of the ultrafiltration fractions: (**A**) DPPH radical scavenging capacity of ultrafiltration fractions. (**B**) ·OH radical scavenging capacity of the ultrafiltration fractions. (**C**) ABTS•+ radical scavenging capacity of the ultrafiltration fractions. ** indicates *p* < 0.01; * indicates *p* < 0.05; ns indicates no significant difference.

Evidence suggests that oxidative stress and dysregulated lipid metabolism are frequently interconnected in metabolic syndrome, and that bioactive peptides exhibiting antioxidant properties may contribute to the amelioration of lipid metabolic disorders. Therefore, in this study, the antioxidant capacity of ultrafiltered peptide fractions derived from CPPH was evaluated by measuring their scavenging activities against DPPH radicals, ·OH radicals, and ABTS•+ radical cations. As shown in Figure 2, a clear trend was observed, wherein the radical scavenging capacity increased with decreasing molecular weight, as indicated by lower IC_50_ values (Figure 2A–C). Note that in Figure 2, lower bars indicate stronger antioxidant activity, as they correspond to lower IC_50_ values. Among the three fractions, the DPPH, ·OH, and ABTS•+ radical scavenging abilities of the CPPH-I fraction were higher than those of the other fractions, with IC_50_ values of 1.510 mg/mL, 0.199 mg/mL, and 3.145 mg/mL, respectively. Notably, the ·OH scavenging ability of CPPH-I was comparable to that of VC, with no significant difference (*p* > 0.05). The results indicated that the CPPH with low-molecular-weight fractions (CPPH-I: <5 kDa) showed the potential to quench the ABTS•+ and DPPH radical species. Consistent with this, Lee et al. reported that the ultrafiltration fractions of bird’s nest peptides with molecular weights less than 5 kDa exhibited high DPPH and ABTS^+^ radical scavenging abilities [29]. It has been suggested that small-molecular-weight bioactive peptides can easily interact with free radicals and interrupt the free radical chain reactions, thereby facilitating antioxidant effects [30]. The antioxidant activity of peptides was more related to the hydrophobic and antioxidant amino acids (including Trp, His, Met, Phe, Tyr, Ala, Pro, Leu, and Gly) in the sequence, as well as their molecular structures [31]. Overall, the fractions with molecular weights of less than 5 kDa exhibited stronger antioxidant activity. Therefore, this study selected this fraction for further separation and purification.

### 3.3. PL-I Activity and Antioxidant Activity of Purified Fractions

**Figure 3:**
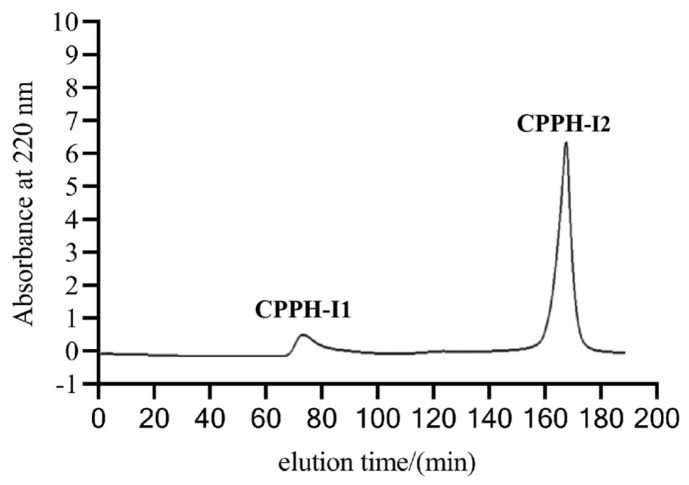
Sephadex G-25 gel chromatogram of CPPH-I.

**Figure 4:**
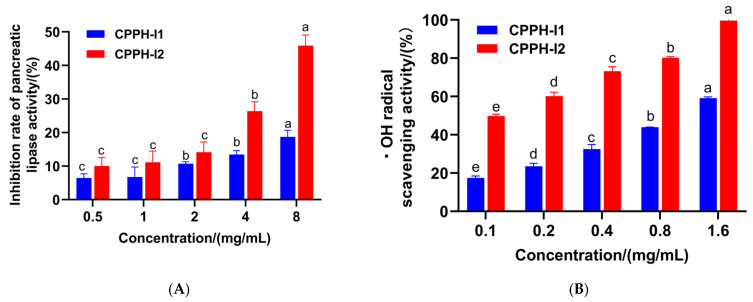
PL-I and antioxidant activities of purified fractions. (**A**) PL inhibition by purified fractions. (**B**) OH radical scavenging rate. Different letters (a–e) above the bars indicate statistically significant differences (*p* < 0.05) among the groups.

To determine the fraction with greater PL-I and antioxidant activities, CPPH-I was separated into two fractions using a gel chromatography column packed with Sephadex G-25 (Figure 3). The PL-I activity and ·OH scavenging activity of the two fractions were determined, and it was found that the PL-I activity of fraction CPPH-I2 was consistently better than that of CHPP-I1 as the concentration increased (Figure 4A). The PL inhibition of CPPH-I2 at a concentration of 8 mg/mL was 45.91 ± 3.17%, which was higher than that of the fraction CPPH-I at the same concentration (39.89 ± 0.54%), suggesting that further purification could significantly enhance the PL-I activity of the peptide.

A similar trend was observed for ·OH radical scavenging activity (Figure 4B). At a peptide concentration of 1.6 mg/mL, fraction CPPH-I2 showed close to near-complete scavenging of ·OH radicals, indicating its strongest antioxidant activity (*p* < 0.05). The IC_50_ of CPPH-I2 was calculated to be 0.122 mg/mL, which is lower than that of the purified fraction. These results are consistent with those of Ketprayoon et al., who reported that the isolated and purified fraction had higher antioxidant and PL-I activities than the ultrafiltration fraction [13]. Therefore, CPPH-I2 was selected for the next step of identification analysis.

### 3.4. Peptide Sequence Identification and Virtual Screening

**Figure 5:**
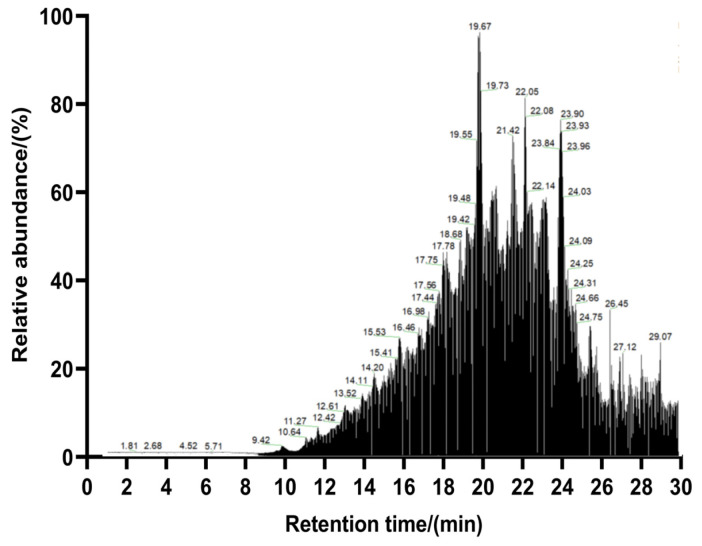
Total ion flow chromatogram of purified peptides.

**Figure 6:**
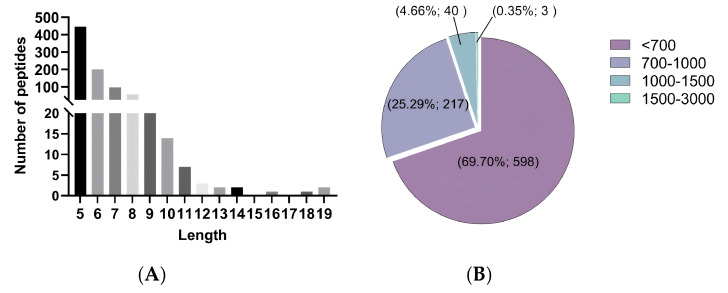
Statistics of the number and length distribution and proportion of peptides. (**A**) Peptide distribution statistics based on amino acid residues. (**B**) Statistics on peptide distribution based on molecular weights.

**Figure 7:**
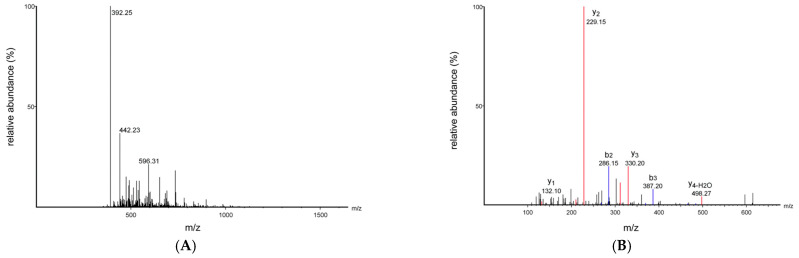
LC-MS/MS analysis of VWTPI. (**A**) MS1 spectrum. (**B**) MS/MS spectrum.

To determine the peptide sequence and molecular weight of the purified peptide from *Chlorella pyrenoidosa*, the peptide sequence in fraction CPPH-I2 (with high PL-I activity) was determined by LC-MS/MS. The overall separation can be reflected by the total ion chromatogram; more and denser peaks indicate a more complex composition of the sample, followed by higher peaks, which usually means a higher content of compounds flowing out at that time point. The larger the peaks, the higher the content of the compounds flowing out of the sample at that time point (Figure 5). A total of 858 peptides were identified in this analysis using PEAKS Studio 8.5 software. The molecular weights of these peptides ranged from 402.20 to 2106.22 Da, with lengths between 5 and 19 amino acids (Figure 6A). The relative contents of pentapeptides, hexapeptides, and heptapeptides were 52.10%, 23.31%, and 25.60%, respectively (Figure 6B). In addition, peptides with relative molecular weights of less than 700 Da accounted for 69.70% of the total peptides, and VWTPI had the highest relative content (Figure 7). Many studies have reported that the bioavailability of peptide drugs decreases significantly when the molecular weight exceeds 700 Da, whereas small peptides composed of 2–6 amino acids with a molecular weight of less than 500 Da may have the highest bioavailability [32]. Therefore, 10 peptides with a high relative content and high identification confidence (−10lgP ≥ 20) were selected as the primary candidates and ranked in descending order of abundance (Table 1).

The ten peptide segments were scored using the peptide activity prediction website PeptideRanker (http://distilldeep.ucd.ie/PeptideRanker/, accessed on 1 August 2025). All peptides received activity scores above 0.5 (Table 2) and were predicted to be soluble and non-toxic. Molecular docking simulations were then performed to further evaluate these ten peptides. A more negative binding energy indicates a stronger interaction between the peptide and PL. Among them, the peptide FLSQPF exhibited the lowest binding free energy (–9.94 kcal/mol), followed by VWTPI (–9.76 kcal/mol), IVGPF (–9.69 kcal/mol), and IPYPL (–9.63 kcal/mol), indicating their potential inhibitory effect on pancreatic lipase (PL) activity. The variation in PL-I activity among these peptides can be attributed to differences in the number and type of amino acid residues within their sequences. A high content of Pro, Leu, and Gly was observed in these PL-inhibitory peptides—a characteristic consistent with known bioactive motifs [33]. Furthermore, hydrophobic amino acids such as Tyr, Leu, Trp, and Phe are typical features of lipid-lowering peptides, as they strongly interact with bile acids, sterols, cholesterol, and other lipids through hydrophobic interactions. Most peptide sequences identified in our current study align with previously reported findings [34]. Therefore, these four peptides (FLSQPF, VWTPI, IVGPF, and IPYPL) were selected for subsequent chemical synthesis and activity validation.

### 3.5. Verification of Activity of Synthetic Peptides

**Figure 8:**
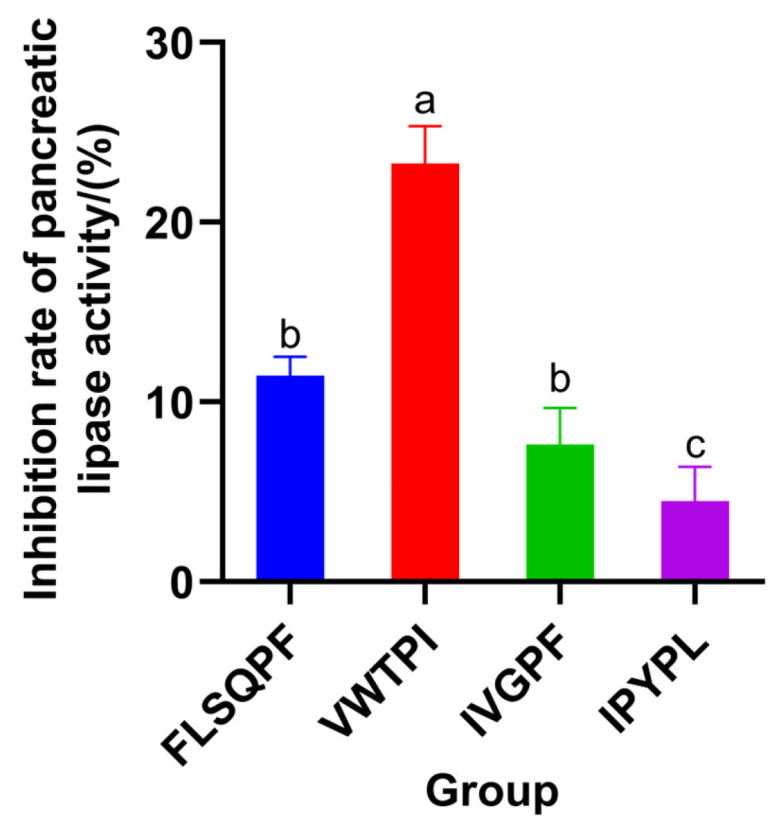
PL-I activity of the synthetic peptides Values of samples bearing different superscript lowercase letters are significantly different (*p* < 0.05).

Database comparisons (BioPepDB, DFBP, and EROP-Moscow) confirmed that no sequences with the same lipid-lowering effect were found among the four peptides. The activity of the synthesized peptides was verified by in vitro experiments, and the four PL-I peptides showed some pancreatic lipase-inhibitory activity at a concentration of 2 mg/mL with inhibition rates of 11.47 ± 1.03%, 23.25 ± 2.08%, 7.64 ± 2.02%, and 4.49 ± 1.89%, respectively (Figure 8). Among them, the pancreatic lipase activity of VWTP was better. It was comparable to that of FDTGSSFYNKPAG in red soybean peptide [35] (36.28% pancreatic lipase inhibition at a concentration of 4 mg/mL), whereas FLSQPF, which had the highest binding energy, had a lower activity, which might be due to the limitations of molecular docking and the solubility and stability of the peptide.

### 3.6. Characterization of the Molecular Interaction Between the Synthetic Peptide VWTPI and PL

To study the possible inhibition pattern of VWTPI on PL, inhibition kinetics were further investigated by the Lineweaver–Burk method. Enzyme inhibition can be classified into reversible and irreversible types of inhibition. The regression curves for the reaction rates were constructed with lipase mass concentration on the x-axis and initial enzymatic reaction rate on the y-axis. A reaction rate profile that intersects with the origin at various inhibitor concentrations suggests reversible inhibition [33]. Reversible inhibition is classified into three types based on its kinetic characteristics: competitive, non-competitive, and mixed inhibition. On the Lineweaver–Burk plots, where the inverse of the reaction rate (1/V) was plotted against the inverse of the substrate concentration (1/[S]), lines intersecting with the vertical axis indicate competitive inhibition; lines intersecting with the horizontal axis indicate non-competitive inhibition; and lines intersecting in the quadrant indicate mixed inhibition [36].

**Figure 9:**
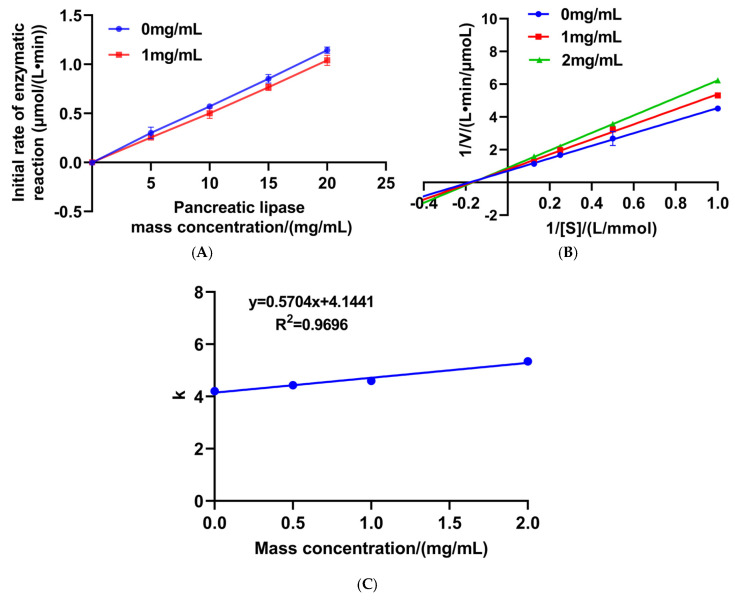
Kinetics of PL inhibition by VWTPI. (**A**) Inhibition mechanism of VWTPI on PL. (**B**) Lineweaver–Burk analysis of VWTPI on PL. (**C**) Determination of the inhibition constant (Ki) of VWTPI.

The regression curves for the reaction rates at different concentrations of VWTPI and varying PL mass concentrations all passed through the origin, confirming that the lipase inhibition mediated by VWTPI is reversible (Figure 9A). The regression models of 1/V (the reciprocal of the reaction rate) and 1/[S] (the reciprocal of the substrate concentration) yielded a set of intersecting lines in the quadrant, indicating that VWTPI exerts mixed competitive inhibition on PL (Figure 9B). This mixed inhibition pattern suggests that VWTPI may bind to both the active site and an allosteric site or interfere with enzyme–substrate complex formation through structural perturbation. This result is consistent with the reported inhibition of sesame protein peptide activity against PL [34].

The inhibition constant (Ki) reflects the affinity between an inhibitor and the enzyme [37]. The calculated Ki value for VWTPI was 7.27 mg/mL (Figure 9C), indicating a moderate binding affinity under in vitro conditions. Nevertheless, these results demonstrate that VWTPI still exhibits detectable inhibitory activity against pancreatic lipase. This study lays the groundwork for the development of functional foods with lipid-lowering potential, and the kinetic results provide preliminary insights into the inhibition mechanism. Further improvements in the pancreatic lipase-inhibitory activity in vitro could be pursued through strategies such as fermentation, peptide structural optimization, and advanced delivery systems.

### 3.7. Molecular Docking Analysis of VWTPI with PL

**Figure 10:**
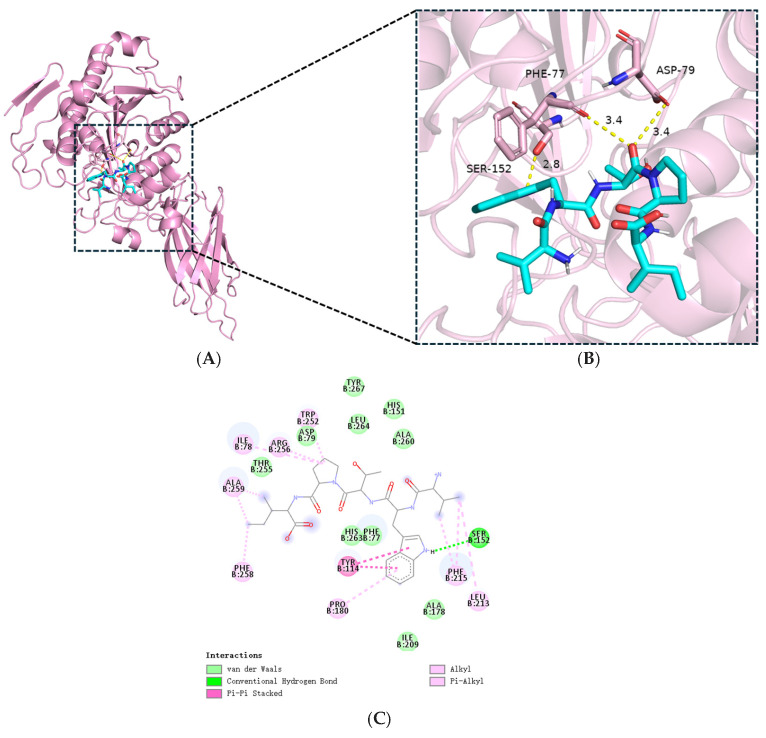
Molecular docking of VWTPI and PL. (**A**) Overall 3D structure of the VWTPI-PL complex. (**B**) Close-up view of the binding pocket. (**C**) Two-dimensional view of the VWTPI-PL interaction.

Figure 10 presents the molecular docking results for the PL-VWTPI complex, revealing optimal binding conformations and sites. Computational analysis predicted that hydrogen bonding, hydrophobic contacts, van der Waals forces, and π-π stacking may contribute to the binding interaction [38]. These interactions involved key residues of the PL catalytic triad (Ser152, Asp176, His263) and substrate binding sites (Phe77, His151, Phe215) (PDB ID: 1LPB). The binding free energy was calculated to be −9.76 kcal/mol. In the predicted model, VWTPI exhibits direct interactions with these crucial residues. Notably, its carbonyl groups were predicted to form hydrogen bonds with Phe77 and with the catalytic residue Ser152 (<2.8 Å) (Figure 10B,C) [39]. Additional interaction included charge-based contacts with His151/His263 and π–π stacking with Phe215. This binding pose suggests that VWTPI may penetrate the hydrophobic cavity of PL, engage the catalytic triad, potentially obstruct substrate access, and thereby align with the observed mixed-competitive inhibition kinetics [40]. This mechanism mirrors that of other reported PL-I peptides like EW, AGY, and QWM, which also target residues within the active pocket or oxyanion hole to block substrate binding [34,40,41]. Therefore, the present study confirms the novel PL-I peptides (VWTPI) in CPPH that inhibit PL activity by occupying catalytic or substrate binding sites.

## 4. Conclusions

In this study, PL-I peptides were isolated and purified from CPPH using ultrafiltration and Sephadex G-25. Four novel PL-I peptides (FLSQPF, VWTPI, IVGPF, and IPYPL) were subsequently identified from the CPPH-I2 fraction for the first time, employing LC-MS/MS coupled with virtual screening. Inhibition kinetics and molecular docking analyses revealed that VWTPI acts as a mixed inhibitor. To date, these specific peptides have not been reported in any natural sources. Therefore, CPPH demonstrates promise as a functional food ingredient to support weight reduction efforts. Future research will focus on evaluating the in vivo lipid-lowering efficacy and exploring formulation strategies to improve peptide stability and bioavailability. These efforts will help clarify the potential of these peptides in future dietary interventions.

## Figures and Tables

**Table 1:** Results of polypeptide sequence identified by LC-MS/MS.

Code	Peptide	RT	Mass	*m*/*z*	Area	−10lgP	Relative Content (%)
1	VWTPI	21.68	614.3428	615.3505	1.40 × 10^9^	29.73	3.36
2	IAYPL	19.37	575.3318	576.3395	1.27 × 10^9^	29.16	3.05
3	IGIML	23.88	545.3247	546.3293	1.17 × 10^9^	26.79	2.81
4	VLPLF	24.16	587.3683	588.3754	9.37 × 10^8^	24.47	2.25
5	FLSQPF	22.21	737.3748	738.3818	9.22 × 10^8^	44.04	2.21
6	GLAPF	19.93	503.2744	504.2817	7.76 × 10^8^	31.03	2.16
7	IVGPF	20.77	531.3057	532.3133	5.92 × 10^8^	33.36	1.86
8	IPYPL	22.16	601.3475	602.3566	5.53 × 10^8^	25.55	1.42
9	YLPPL	21.46	601.3475	602.3548	5.37 × 10^8^	23.73	1.33
10	SYLPPL	22.02	688.3795	689.387	5.01 × 10^8^	44.30	1.29

**Table 2:** Molecular docking energy between peptides and PL.

Code	Peptide	Affinity (kcal/mol)	PeptideRanker Score	Solubility	Toxicity
1	FLSQPF	−9.94	0.527	Good	Non-Toxic
2	VWTPI	−9.76	0.636	Good	Non-Toxic
3	IVGPF	−9.69	0.702	Good	Non-Toxic
4	IPYPL	−9.63	0.816	Good	Non-Toxic
5	SYLPPL	−9.50	0.853	Good	Non-Toxic
6	YLPPL	−9.28	0.908	Good	Non-Toxic
7	VLPLF	−9.03	0.793	Good	Non-Toxic
8	GLAPF	−9.03	0.850	Good	Non-Toxic
9	IGIML	−8.82	0.870	Good	Non-Toxic
10	IAYPL	−8.70	0.853	Good	Non-Toxic

## Data Availability

All relevant data are included in the article. Further inquiries can be directed to the corresponding author.

## Abbreviations

The following abbreviations are used in this manuscript.

CPPH	*Chlorella pyrenoidosa* Protein Hydrolysate
PL	Pancreatic Lipase
PL-I	Pancreatic Lipase—Inhibitory
